# Comparative assessment of birth preparedness and complication readiness among couples in rural and urban communities of Ekiti State, Southwestern Nigeria

**DOI:** 10.4314/gmj.v58i1.6

**Published:** 2024-03

**Authors:** Ademuyiwa Adetona, Olusegun E Elegbede, Olusola O Odu, Kabir A Durowade, Tope M Ipinnimo, David S Ekpo, Taofeek A Sanni

**Affiliations:** 1 Department of Community Medicine, Afe Babalola University, Ado-Ekiti, Nigeria; 2 Department of Community Medicine, Federal Teaching Hospital, Ido-Ekiti, Nigeria; 3 Department of Community Medicine, Ekiti State University Teaching Hospital, Ado Ekiti, Nigeria

**Keywords:** Birth preparedness, complication readiness, couples

## Abstract

**Objectives:**

To assess and compare the level of Birth Preparedness and Complications Readiness (BPCR) and determine the predicting effect of socio-demographic factors on it among couples in rural and urban communities of Ekiti State.

**Design:**

A community-based comparative cross-sectional study.

**Setting:**

The study was conducted in twelve rural and twelve urban communities in Ekiti State.

**Participants:**

Couples from rural and urban communities. Female partners were women of reproductive age group (15-49 years) who gave birth within twelve months before the survey.

**Main outcome measures:**

Proportion of couples that were well prepared for birth and obstetric emergencies, and its socio-demographic determinants.

**Results:**

The proportion of couples that were well prepared for birth and its complications was significantly higher in urban (60.5%) than rural (48.4%) communities. The study also revealed that living above poverty line (95% CI=1.01–3.79), parity and spousal age difference less than five years (95% CI=1.09 – 2.40) were positive predictors of BPCR among respondents.

**Conclusions:**

Urban residents were better prepared than their rural counterparts. Living above poverty line, parity, and spousal age difference less than five years were positive predictors of BPCR. There is a need to emphasize on educating couples on the importance of identifying blood donors as a vital component of BPCR.

**Funding:**

None declared

## Introduction

Giving birth to a child is an event of happiness that is celebrated globally, and people in sub-Saharan Africa (SSA) place such a high premium on having children that women who do not give birth to children are usually looked down upon in the society.^1^ However, pregnancy and childbirth may be associated with some risks which may result in maternal and newborn morbidity and mortality.^2^ Delay in access to quality emergency obstetric care has been identified as one of the determinants of preventable maternal deaths.^3^

The strategy of BPCR aims to increase the timely use and effectiveness of key services for mothers and newborns, particularly during childbirth.^4^

Most couples rarely plan for births because they believe that pregnancy and childbirth are natural events that are expected to result in normal outcomes.^5^ The outcome of pregnancy and its sequelae are usually left to divine providence, most especially in rural communities.^6^ Majority of Nigerian women live in rural communities where the burden of maternal ill-health is higher relative to urban communities.^6^ The strategy of BPCR is comprised of principles that encourage women and families to make decisions before the onset of labour in case of obstetric complications.^7^

Pregnancy, childbirth and complications are the leading causes of mortality among women of reproductive age groups in developing countries.^8^

About two-thirds (66%) of global maternal deaths occurred in SSA, and there are large disparities between rural and urban communities with more maternal deaths occurring in rural communities.^9^,^10^ These deaths are mostly attributed to mothers' lack of knowledge of antenatal care (ANC) services, birth preparedness, and obstetric danger signs.^11^ Most of these deaths could have been prevented with adequate, timely, and quality care.

Regrettably, a large proportion of women in Nigeria still lose their lives to pregnancy and childbirth-related complications.^9^ Although BPCR is essential for further improvement of maternal and child health, little is known about the knowledge and practice of this strategy by pregnant women and their spouses in Nigeria.^2^ The estimated MMR for Nigeria is 512/100000 live births, with 67000 maternal deaths accounting for 23% of maternal deaths globally.^9^,^12^ In Nigeria, MMR remains unacceptably high due to ineffective implementation of the BPCR strategy.^4^ Gaps exist between urban and rural areas, with more maternal deaths occurring in rural areas.^8^

In patriarchal societies, including Nigeria, pregnancy and childbirth are usually regarded as women's affairs, and only a few men accompany their wives to ANC.^5^,^13^ This is the reason why men have little knowledge when it comes to the issue of BPCR, which is usually taught during ANC visits. However, cultural and religious factors subject women to depend on their husbands. Men are socially and economically dominant and they mostly determine their wives' access to healthcare.^5^,^14^ This implies that men should be included in interventions to reduce maternal mortality to achieve the desired outcome. The benefits of BPCR are likely to be optimized when it is undertaken as a joint process between women and their spouses.^15^

Rural communities represent highly marginalized areas in terms of distribution and access to healthcare interventions, including maternal and child health services. This underscores the need to examine the influence of the location of residence and other factors associated with the level of BPCR. Consequently, the study attempted to provide useful information on the comparison between rural and urban communities in terms of their level of preparedness for childbirth and obstetric complications and the associated socio-economic factors in Ekiti State.

## Methods

### Study area

The study was conducted in Ekiti State, which has 16 Local Government Areas (LGAs), of which 10 were classified as urban and 6 as rural. The majority of the residents of rural areas are farmers and petty traders, while other occupations such as civil service, trading, organized private sector, commercial driving, and artistry are concentrated in the urban communities.

At a projection of 3.15% per year the state had a population of 3,939,597 in 2020 with the urban being more densely populated than the rural areas (National Population Commission (NPC), 2006). The primary frontline health facilities in the rural LGAs are primary and secondary health facilities while the urban LGAs have primary, secondary and tertiary hospitals with Ado-Ekiti LGA having two Teaching Hospitals. Antenatal and delivery services are provided in all the health facilities. The rural LGAs have secondary schools while the urban LGAs have tertiary institutions as highest educational facilities.

### Study design and participants

This community-based cross-sectional study was conducted among women of reproductive age group and their male partners. A total of 520 couples with equal representation from rural and urban communities participated in the study. The eligibility criteria for recruitment for the study included the following: being a permanent resident in the study areas; willingness to participate in the survey; women of reproductive age group (15 – 49 years) who gave birth within 12 months before the study regardless of their birth outcome, and their male partners.

### Sample Size Determination

The minimum sample size was determined using the formula for comparing two proportions:^16^


$$n=\frac{\left(U+V{)}^{2}[P_{1}(100-P_{1})+P_{2}(100-P_{2})\right]}{(P_{1}-P_{2}{)}^{2}}$$

Where:

n = Minimum sample size per group

U= Standard normal deviate corresponding to the power of 80% = 0.84

V= Standard normal deviate corresponding to confidence level of 95% for two-tailed test = 1.96

Using the proportion of women that were well prepared (P_1_=61 %) from a previous study in an urban city in southern Nigeria,^17^ and a lower proportion of women that were well prepared (P_2_=48.4%) in a rural community in southern Nigeria,^18^ and compensating for 10% non-response rate, the sample size was adjusted to a total of 260 couples per group.

### Sampling technique

Participants were recruited using a multi-stage sampling techniqu**e.** In stage 1, two rural and two urban LGAs were selected using a simple random sampling technique by balloting. In stage 2, two communities were selected from each of the selected rural and urban LGAs using table of random numbers. In stage 3, three enumeration areas (EAs) were selected from each of the eight communities by balloting, making a total of twenty-four EAs (12 in rural and 12 in urban communities).

With the aid of EA maps from the NPC of Nigeria, we were able to determine the boundaries of the selected EAs. Household numbering was done in each of the selected EAs, and houses with eligible partners were mapped out. A total of 528 and 660 eligible households were listed in the rural and urban areas, respectively. Since the equal allocation of the sample size was apportioned to each of the selected EAs, sampling intervals of two and three for rural and urban areas, respectively, were calculated by dividing the total number of households in each group by the sample size of 260. In stage 4, systematic random sampling was applied to select the households with eligible couples to be interviewed. After randomly selecting the first household in each EA by picking any number within the sampling interval, subsequent households were selected by adding the sampling interval to the selected number until the sample size was attained.

### Data collection

A semi-structured pretested interviewer-administered questionnaire adopted from a standardized questionnaire on BPCR developed by John Hopkins Program for International Education in Gynecology and Obstetrics was used to collect the data over a period of thirteen weeks.^9^ It was designed to seek information about the couple's socio-demographic characteristics, level of BPCR as well as the associated factors. Data were collected from eligible couples in the privacy of their homes through face-to-face interviews after obtaining their consent. The couples were interviewed individually at the same time.

### Data analysis

Collected data were analyzed using IBM SPSS version 24. Categorical variables were summarized as proportions and compared between rural and urban communities. Quantitative variables were summarized as means ± standard deviation and compared between rural and urban communities. Bivariate analysis was done to determine the significance of the associations between the dependent variable BPCR and independent socio-demographic variables using Pearson's chi-square test. All significant socio-demographic variables were then included in a binary logistic regression model to determine their independent effects.

Couples who met at least four of the following eight BPCR criteria: saving money towards delivery, ANC attendance, identifying a place for delivery, identifying a birth companion, planning for transportation, identifying a blood donor, husband accompanying wife to ANC and delivery in a health facility were considered well prepared while those who met less than four criteria were considered poorly prepared.^18^

Zero score was awarded when there was no concordance and a score of one when there was concordance in at least four of the BPCR criteria between a woman and her partner. The poverty line was fixed at $1.90 per day.^19^ A p-value of <0.05 was taken as statistically significant.

### Ethical approval

Ethical approval was obtained from the Ethics and Research Review Committee of the Federal Teaching Hospital, Ido-Ekiti, Nigeria (reference number ERC/2018/07/10/127B), and written informed consent was obtained from the participants.

## Results

Table 1 shows the socio-demographic characteristics of the study participants. A total of 520 couples with equal proportions from rural and urban communities participated in the study. The women and their partners in urban communities were significantly older than those in the rural areas. The mean age of women from the rural communities was 29.9±6.5 years while that of those from urban communities was 31.0±5.6 years (p=0.039). Also the mean age of men in urban areas was 36.8±5.7 years while that of those from the rural areas was 35.5±7.0 years (p=0.015). Most of the respondents were educated with a significantly higher proportion of urban respondents (women 172(66.2%), men 168(64.6%), p=0.001) having tertiary education compared to their rural counterparts (women 86 (32.9%), men 89(34.1%), p=0.001). More than four-fifths 217(83.5%) of urban couples were living above poverty line compared to rural group 113(43.4%), (p<0.001).

**Table 1:** Socio-demographic characteristics of couples in rural and urban communities

Variable	Area		
	**Rural**	**Urban**	**p-value**
	**n (%) = 260**	**n (%) = 260**	
Women's Age Group (in years)			
**Less than 20**	8 (3.1)	4(1.5)	0.197
**21** – **29**	101 (38.8)	88 (33.8)	
**30** – **39**	131 (50.4)	153 (58.8)	
**40 and above**	20 (7.7)	15 (5.9)	
***Mean age ± SD***	*29.9 ± 6.5*	*31.0± 5.6*	***0.039***
Men's Age Group (in years)			
**21** – **29**	53 (20.5)	26 (10.0)	**0.004**
**30** – **39**	117 (45.0)	135 (51.9)	
**40 and above**	90 (34.5)	99 (38.1)	
***Mean age ± SD***	*35.5 ± 7.0*	*36.8± 5.7*	***0.015***
Spousal Age Difference (in years)			
**Less than 5**	109 (41.9)	98 (37.7)	0.765
**5** – **9**	102 (39.1)	113 (43.5)	
**10** – **14**	37 (14.1)	37 (14.2)	
**15 above**	12 (4.7)	12 (4.7)	
***Mean age difference ± SD***	*6.0± 4.8*	*6.5 ± 4.3*	*0.286*
Women's Highest Educational Level	5 (1.9)	0 (0.0)	**0.001**
**None**			
**Primary**	26 (10.1)	8 (3.1)	
**Secondary**	143 (55.0)	80 (30.8)	
**Tertiary**	86 (32.9)	172 (66.2)	
**Men's Highest Educational Level**			
**None**	5 (1.9)	2 (0.8)	**0.001**
**Primary**	25 (9.7)	6 (2.3)	
**Secondary**	141 (54.3)	84 (32.3)	
**Tertiary**	89 (34.1)	168 (64.6)	
Average monthly income			
**Below Poverty line**	147 (56.6)	43 (16.5)	**<0.001**
**Above poverty line**	113 (43.4)	217 (83.5)	

Table 2 shows the complications experienced by the women in the last pregnancy. The majority of the rural women significantly experienced high fever, 51(37.0%), while only about one-fifth, 24(18.9%) of their urban counterparts experienced it (p<0.001). The complication experienced most among urban women was severe headache 37(29.1%), which occurred in a higher proportion 44(31.9%) among rural women (p=0.627). The majority of the women sought assistance for these problems (rural 107(79.0%), urban 118(92.9%), p<0.001) from SBAs (p=0.012).

**Table 2:** Complications experienced during the current pregnancy among women who gave birth in the last 12 months

Variable	Area		
	Rural n (%)	Urban n (%)	p-value
Problems experienced during the current pregnancy*	***n=138***	***n =127***	
**Bleeding**	41 (29.7)	20 (15.7)	**0.007**
**Severe Headache**	44 (31.9)	37 (29.1)	0.627
**Blurred Vision**	36 (26.1)	20 (15.7)	**0.039**
**Convulsions**	31 (22.5)	12 (9.4)	**0.004**
**Swollen Hands/Face**	39 (28.3)	27 (21.3)	0.188
**High Fever**	51 (37.0)	24 (18.9)	**0.001**
**Loss of Consciousness**	34 (24.6)	15 (11.8)	**0.007**
**Breathing Difficulty**	35 (25.4)	15 (11.8)	**0.005**
**Severe Weakness**	46 (33.3)	25 (19.7)	**0.012**
**Severe Abdominal Pain**	48 (34.8)	26 (20.5)	**0.009**
**Reduced Fetal Movement**	40 (29.0)	13 (10.2)	**0.001**
**Water Breaks Without Labor**	39 (28.3)	16 (12.6)	**0.002**
Who made the final decision about whether or not to seek assistance for this problem	***n=138***	***n =127***	
**Respondent**	15 (10.9)	14 (11.0)	0.917
**Respondent & husband**	89 (64.5)	79 (62.2)	
**Husband**	34 (24.6)	34 (26.8)	
Sought assistance for the problem	***n=138***	***n =127***	
**Yes**	109 (79.0)	118 (92.9)	**0.001**
**No**	29 (21.0)	9(7.1)	
Reasons for not seeking assistance for the problem	***n = 29***	***n = 9***	
**You didn't think it is necessary**	12 (41.4)	4 (44.4)	0.786^LR^
**Husband/family didn't think it necessary**	5 (17.2)	2 (22.2)	
**Health Facility too far**	4 (13.8)	0 (0.0)	
**No transport**	1 (3.4)	0 (0.0)	
**Too expensive**	1 (3.4)	1 (11.2)	
**Home remedy**	6(20.8)	2 (22.2)	
Who was seen for assistance for the problem	***n = 109***	***n = 118***	
**Doctor**	47 (43.1)	79 (63.1)	**0.012**
**Nurse/Midwfe**	39 (35.7)	28 (27.1)	
**TBA**	5 (4.6)	0 (0.0)	
**Community Health Worker**	15 (13.8)	10 (8.5)	
**Relative/Friend**	3 (2.8)	1 (0.8)	
Went to health facility for assistance	***n = 109***	***n = 118***	
**No, did not go**	27 (24.8)	6 (5.1)	**<0.001**
**Government hospital**	34 (31.2)	61 (51.6)	
**Government health centre**	39 (35.8)	35 (29.7)	
**Private clinic**	9 (8.3)	16 (13.6)	

* Multiple responses, LR= likelihood ratio

Table 3 shows most of the women who attended ANC were being attended to by SBAs (rural: 248(97.8%), urban: 254(98.4%), p=0.001). However, only a few husbands accompanied their wives to ANC (rural: 70(27.4%), urban: 94(36.5%), p=0.115). Identifying potential blood donors was also significantly low, with the proportion of the urban population being about twice 87(33.9%), and that of the rural population was 44(17.4%) couples (p=0.002). Most of the deliveries were conducted in government health facilities, with the urban communities having a higher proportion (rural 202(79.7%), urban 211(81.9%), p=0,001). Generally, more couples in the urban 156(60%) were significantly well prepared for birth and its complications than those in rural 126(48.4%) communities (p=0.039).

**Table 3:** Comparative assessment of birth preparedness and complication readiness among couples in rural and urban communities

Variable	Area		
	Rural n (%) N = 260	Urban n (%) N = 260	p-value
Attended ANC during the pregnancy			
**Yes**	254 (97.7)	258 (99.2)	0.355
**No**	6 (2.3)	2 (0.8)	
First attended to during a checkup by	***n=254***	***n =258***	
**Doctor**	40 (15.6)	86 (33.3)	**0.001**
**Nurse/ Midwife**	105 (41.5)	125 (48.4)	
**TBA**	6 (2.2)	4 (1.6)	
**Community Health Worker**	103 (40.7)	43 (16.7)	
Identified a place of delivery	***n=254***	***n =258***	
**Yes**	225 (88.4)	242 (93.7)	0.216
**No**	29 (11.6)	16 (6.3)	
Made arrangements for transport	***n=254***	***n =258***	
**Yes**	175 (68.8)	201 (78.0)	0.094
**No**	79 (31.2)	57 (22.0)	
Made arrangements for finances/funds	***n=254***	***n =258***	
**Yes**	215 (84.8)	232 (89.8)	0.226
**No**	39 (15.2)	26 (10.2)	
Identified a potential blood donor	***n=254***	***n =258***	
**Yes**	44 (17.4)	87 (33.9)	**0.002**
**No**	210 (82.6)	171 (66.1)	
Husband accompanied wife to ANC	***n=254***	***n =258***	
**Yes**	70 (27.4)	94 (36.5)	0.115
**No**	184 (72.6)	164 (63.5)	
Identified a birth companion	***n=254***	***n =258***	
Yes	**142 (55.8)**	**149 (57.9)**	**0.654**
No	**112 (44.2)**	**109 (42.1)**	
Where delivery was taken	***n=254***	***n =258***	
Government hospital	**42 (16.7)**	**120 (46.5)**	**0.001**
Government health centre	**160 (63.0)**	**91 (35.4)**	
Private clinic	**24 (9.4)**	**33 (12.6)**	
Home	**4 (1.4)**	**4 (1.6)**	
TBA	**24 (9.4)**	**10 (3.9)**	
Well prepared	126 (48.4)	156 (60.0)	**0.039**
Poorly prepared	134 (51.6)	104 (40.0)	

Overall, spousal age difference, women's educational level, couple's monthly income and women's parity were significantly associated with BPCR. The spousal age difference was a significant factor in urban (χ^2^=7.903, p=0.048) but not in rural (χ^2^=3.314, p=0.346) communities. Couples with lower age differences were less well prepared for birth and its complications than those with higher age differences in rural communities. Conversely, the urban group did not follow the same trend, with those with an age difference of 5-9 years having the highest proportion (73.5%) of those who were well prepared. In addition, the educational status of women is a statistically significant factor in rural but not in urban communities (rural: χ^2^=10.067, p=0.018 and urban: χ^2^=5.096, p=0.078). Couples that were well-prepared in both communities were mostly those with educated women.

Monthly income is a statistically significant factor in rural but not in urban communities (χ^2^=5.474, p=0.019 and χ^2^=0.472, p=0.492, respectively).

A significantly higher proportion of couples living above 78(69.1%) compared to those living below 79(53.7%) the poverty line in rural communities were well prepared for birth and its complications (p=0.019), but the reverse is the case among urban couples with those living below the poverty line having higher proportion (below poverty line: 46(70.8), above poverty line: 129(66.2%), p=0.492).

Parity is a significant factor associated with BPCR in both rural and urban communities (χ^2^=14.467, p=0.006 and χ^2^=16.335, p=0.003, respectively). In this study, there is an inverse relationship between the level of BPCR and parity. The lower the parity of female partners, the more likely couples are to be well prepared. Urban couples were generally better prepared for births in relation to the parity of female partners except for para 5 and above, where rural communities had a higher proportion of 12(36.4%) of couples that were well prepared than urban communities 5(29.4%) (Table 4).

**Table 4:** Comparative assessment of birth preparedness and complication readiness by socio-demographic characteristics among couples in rural and urban communities

Variable	Rural			Urban		

	Practice of birth preparedness and complication readiness		Total N=260	Practice of birth preparedness and complication readiness		Total N = 260
	
	Well prepared n (%)	Poorly prepared n (%)		Well prepared n (%)	Poorly prepared n (%)	
Spousal Age Difference (in years)						
**Less than 5**	60 (54.6)	49 (45.4)	109	67 (68.4)	31 (31.6)	98
**5** – **9**	59 (57.4)	43 (42.6)	102	83 (73.5)	30 (26.5)	113
**10** – **14**	25 (67.6)	12 (32.4)	37	19 (51.4)	18 (48.6)	37
**15 and above**	9 (75.0)	3 (25.0)	12	6 (50.0)	6 (50.0)	12
***Statistics***	*χ^2^ = 3.314*,	*p= 0.346*		χ*^2^ = 7.903*,	*p = **0.048***	
Men's Highest Educational Level						
**None**	2 (40)	3 (60)	5	0 (0.0)	2 (100.0)	2
**Primary**	16 (64.0)	9 (36)		2 (33.3)	4 (66.7)	6
**Secondary**	83 (58.6)	58 (41.4)	25	59 (70.2)	25 (29.8)	84
**Tertiary**	52 (58.0)	37 (42.0)	141	114 (67.9;	54 (32.1)	168
***Statistics***	*χ^2^* = 1.028,	*p = 0.795*	89	*χ^2^ = 7.616*,	*p = 0.055*	
Women's Highest Educational Level						
**None**	1 (20.0)	4 (80.0)	5	-	-	-
**Primary**	15 (57.7)	11 (42.3)	26	3 (37.5)	5 (62.5)	8
**Secondary**	75 (52.8)	67 (47.2)	143	50 (62.5)	30 (37.5)	80
**Tertiary**	61 (70.6)	25 (29.4)	86	122 (70.9)	50 (29.1)	172
***Statistics***	*χ^2^ = 10.067*,	**p= 0.018**		*χ^2^ =5.096*,	p = 0.078	
Average monthly income						
**Below Poverty line**	79 (53.7)	68 (46.3)	147	46 (70.8)	19 (29.2)	65
**Above poverty line**	78 (69.1)	35 (30.9)	113	129 (66.2)	66 (33.8)	95
***Statistics***	*χ^2^ = 5.474*,	**p = 0.019**		*χ^2^ =0.472*,	p=0.492	
Women's parity						
**1**	10 (90.9)	1 (9.1)	11	8 (100.0)	0 (0.0)	8
**2**	67 (62.9)	39 (37.1)	106	80 (72.1)	31 (27.1)	111
**3**	44 (63.9)	25 (36.8)	69	56 (65.1)	30 (34.9)	86
**4**	20 (48.8)	21 (51.2)	41	26 (68.4)		38
**5 and above**	12 (36 4)	21 (63.6)	33	5 (29.4)	12 (70.6)	17
***Statistics***	*χ^2^ = 14.467*,	**p = 0.006**		*χ^2^ =16.335*,	**p = 0.003**	

Table 5 shows the binary logistic regression for socio-demographic predictors of BPCR. Couples with low spousal age differences are more likely to be well-prepared. Spousal age differences of less than 5 years are significant predictors of being well-prepared in the urban community (AOR=1.28, 95% CI=1.09-2.40). Also, couples living above the poverty line were about twice as likely to be well prepared for birth and its complications than those living below the poverty line in rural communities (AOR=1.96, 95% CI=1.01-3.79).

**Table 5:** Binary logistic regression for the predictors of good practice of birth preparedness and complication readiness among couples in both rural and urban communities

Variable	Rural		Urban	
	AOR (95% CI)	p-value	AOR (95% CI)	p-value
Spousal Age Difference (in years)				
**Less than 5**	-		**1.283 (1.086** – **2.399)**	**0.032**
**5** – **9**	-		0.501 (0.218 – 1.154)	0.365
**10** – **14**	-		0.264 (0.065 – 1.069)	0.619
**15 above**	-		1.000	
Tribe				
**Yoruba**	1.000			
**Hausa**	0.125 (0.014 – 1.091)	0.083		
**Igbo**	0.610 (0.176 – 2.116)	0.630		
**Others**	0.6/8 (0.180 – 2.551)	0.646		
Highest Educational Level				
**None**	1.000		-	
**Primary**	6.739 (0.545 – 83.326)	0.155	-	
**Secondary**	3.602 (0.334 – 38.865)	0.309	-	
**Tertiary**	5.192 (0.466 – 57.807)	0.116	-	
Average monthly income				
**Below Poverty line**	1.000			
**Above poverty line**	**1.958 (1.013** – **3.785)**	**0.039**		
Women's parity				
**1**	**21.149 (2.230** – **200.568)**	**0.009**	**6.524 (1.897** – **25.698)**	**0.002**
**2**	**2.774 (1.149** – **6.697)**	**0.034**	**5.438 (1.616** – **18.299)**	**0.007**
**3**	**2.670 (1.041** – **6.851)**	**0.048**	**3.818 (1.137** – **12.822)**	**0.032**
**4**	1.721 (0.628 – 4.713)	0.303	**4.174 (1.092** – **15.951)**	**0.042**
**5 and above**	1.000		1.000	

AOR: Adjusted Odd Ratio; CI: Confidence Interval

Parity was a significant predictor in both rural and urban communities as primipara were 21 times (AOR=21.15, 95% CI=2.23–200.57) more likely to be well prepared than their counterparts with at least para 5 in rural compared to about 7 times (AOR=6.52, 95% CI=1.90-25.70) in urban communities. Para 2 and para 3 are positive predictors of the level of BPCR in both rural and urban communities, while para 4 is a positive predictor only in urban communities.

## Discussion

This study assessed and compared the practice of BPCR among couples residing in rural and urban communities of Ekiti State. The women and their partners in the urban areas were older than those in the rural areas probably because, as shown in the study, urban men and women were more educated than their rural counterparts, particularly in the attainment of tertiary education. People in tertiary schools will likely want to complete their education before getting married, thereby making them marry late compared to their age mates who are not educated or partially educated and who would have married earlier.

About half (48.4%) of rural couples, compared to two-thirds (60.0%) of urban couples, were well prepared for birth and its complications. The level of BPCR among the urban couples in this study was higher, probably due to better earning capacity and being more educated than their rural counterparts. These results are similar to what have been reported in other studies. A similar study done in northern Nigeria reported 94.1% among urban compared to 78.2% among rural respondents.^20^ Similarly, a study conducted in Benin an urban city in southern Nigeria reported 87.4%.^21^

The level of preparedness in some of the studies above were higher than the findings in this study because they were conducted among pregnant women attending ANC probably following sessions during which they would have been counseled on BPCR. However, some studies reported that less than 50.0% of respondents were well prepared for birth and its complications. Some of these studies conducted in Nigeria, Uganda, Ethiopia, India, Tanzania, and Ghana reported 48.4%,^18^ 35.0%,^22^ 27.5%,^23^ 47.8%,^24^ 58.4%^25^ and 46.5%^26^ respectively.

The difference in the level of preparedness between this study and the others above could also be related to the difference in means of measurements used to determine the level of BPCR and sociocultural differences. However, a study conducted in rural Bangladesh using the same means of measurements to determine the level of BPCR reported only 10% of the participating couples were well prepared.^15^ This very low level of preparedness reported in the study compared to ours may be due to illiteracy, where the majority of the couples either had no formal education or had only primary education.

Identification of potential blood donors by couples prior to delivery helps to mitigate obstetric complications that may warrant blood transfusion. However, the majority of the respondents in this study did not have any form of preparation for emergency blood transfusion. This is similar to the findings of studies in southeast^27^ Nigeria but in contrast to a study done in Osogbo southwest Nigeria, which reported 60.8%.^28^

The findings in this study are also consistent with studies conducted in Ghana,^29^ Ethiopia,^30^ Tanzania,^25^ India^31^ and Bangladesh.^15^ The low level of preparation in identifying potential blood donors prior to delivery in this study may be due to the ignorance of the respondents on the importance of preparing for an emergency or obstetric complications that may arise in pregnancy and during delivery.

There was a low level of ANC attendance by men in this study; 36.5% of male respondents were in urban areas, compared to 27.4% in rural communities. These are in consonance with studies done in northern Nigeria (32.1%)^7^ and Nepal (39.3%).^32^ In a patriarchal society like Nigeria, ANC is regarded as women's affairs,^5^ and this may also contribute to low male ANC attendance in this study. In contrast, studies conducted in Uganda^22^ and Ethiopia^33^ reported high levels of male ANC attendance (65.4% and 61.9%, respectively).

In this study, it was found that the socio-demographic characteristics of the respondents were significantly associated with the level of BPCR. The educational status of couples played a significant role in their level of BPCR in this study. Educated women have the capability of deciding on issues related to their health, and they also have the ability to better understand health messages and search for more information regarding health issues.^5^ Therefore, education enhanced their health-seeking action and level of BPCR. Most of the respondents in this study had a certain degree of education. This is consistent with findings from similar studies in northern Nigeria,^4^ Uganda^34^ and India.^35^

High socioeconomic status may be associated with better practice of BPCR. Higher household income may lead to increased utilization of skilled maternal health services.

The parity of female respondents is another significant factor in this study. This had an inverse relationship with the level of preparedness, and it was similar to studies done in Benin City, Nigeria,^36^ Ethiopia^23^ and India.^37^ A primipara would likely be well prepared compared to a woman with higher parity because she lacked previous experience of such. She would probably be more cautious about her pregnancy and, therefore, seek an SBA who would educate her on BPCR. The higher proportion of urban respondents that were well prepared compared to their rural counterparts in this study may be ascribed to inequities in the number of accessible health facilities.^38^

Respondents were required to remember information retrospectively, recall bias and lack of agreement between the couples concerning what they did in preparation for birth were the limitations of this study.

## Conclusion

Our findings showed that more **couples** in urban than rural communities were well prepared for birth and its complications, but male involvement in ANC attendance was low in this study. Moreover, only a few couples identified potential blood donors in preparation for any complication of birth that may warrant blood transfusion. A spousal age difference of less than five years and parity were the determinants of BPCR in urban communities, while living above the poverty line and parity were determinants of BPCR in rural communities.
